# A Dual-Scale Encapsulation Strategy for Phase Change Materials: GTS-PEG for Efficient Heat Storage and Release

**DOI:** 10.3390/nano15241887

**Published:** 2025-12-16

**Authors:** Sixing Zhang, Guangyao Zhao, Zhen Li, Zhehui Zhao, Jiakang Yao, Geng Qiao, Zongkun Chen, Yuwei Wang, Donghui Zhang, Dongliang Guo, Zhixiang Zhu, Yu Han

**Affiliations:** 1 State Key Laboratory of Advanced Power Transmission Technology, China Electric Power Research Institute Co., Ltd., Beijing 102209, China; zhangsixingsgcc@163.com (S.Z.);; 2 Global Energy Interconnection Research Institute Europe GmbH, 10623 Berlin, Germany; 3 Beijing Guodian Futong Science and Technology Development Co., Ltd., Beijing 100070, China; 4 State Grid Jiangsu Electric Power Co., Ltd., Research Institute, Nanjing 211103, China

**Keywords:** phase change materials, framework, thermal conductivity, energy conversion

## Abstract

With the advancement of new power systems, phase-change materials (PCMs), owing to their ability to convert and store electrical energy, are increasingly recognized as a key solution to the intermittency of power supply. Nevertheless, such materials face challenges, including leakage and low thermal conductivity, which lead to reduced utilization efficiency. In this study, guar gum was used as the macroscopic framework, while self-prepared and optimized silica aerogel microsheets served as the microscopic framework to synergistically encapsulate the polyethylene glycol (PEG). Titanium dioxide (TiO_2_) nanoparticles were incorporated to improve overall thermal conductivity, resulting in the composite PCM, GTS-PEG. In-depth characterization demonstrated effective PEG retention within the matrix, with a melting heat storage density of 164.16 J/g. Upon 30 min of continuous heating at 90 °C, the mass loss remained as low as 4.83%, indicating excellent thermal stability. The addition of TiO_2_ increased thermal conductivity to 0.53 W/(m·K), representing a 140% boost over unmodified material. As a result, GTS-PEG not only successfully overcomes the leakage and thermal conductivity limitations of conventional PCMs but also, as a green and low-carbon innovative solution, paves a new path for the coordinated optimization and efficient conversion of power grid energy systems.

## 1. Introduction

The new-type power system refers to a configuration primarily based on new energy sources, in which intelligent and digital technologies are employed to achieve efficient coordination and flexible regulation across energy production, transmission, and consumption [1,2,3]. Nonetheless, the new-type power system faces serious imbalances between electric energy distribution and load demand, along with the inherent intermittency and volatility of renewable energy [4,5,6]. Under such circumstances, the demand for large-scale, long-duration energy storage across the generation, grid, and load sides is particularly urgent. On the generation side, high-capacity energy storage is able to effectively mitigate power fluctuations from wind and solar sources, enhance integration capacity of renewable energy, support reliable operation of multi-energy complementary systems, and promote both large-scale transmission of offshore wind power and local consumption of distributed renewable energy [7,8]. On the grid side, long-duration, large-capacity energy storage has the capacity to fulfill peak shaving, frequency regulation, and voltage control functions, significantly improving the resilience, security, and stability of the power grid [9]. On the load side, with the widespread integration of photovoltaics leading to midday load valleys, long-duration energy storage is capable of contributing to peak shaving and valley filling, balancing supply and demand, and functioning as an “energy regulation reservoir” capable of storing energy across diurnal cycles, thereby ensuring the continuous and stable operation of the power system [10,11].

Therefore, the development of long-duration, large-capacity energy storage is essential for addressing the challenges of the power system and facilitating energy transition [12,13]. Emerging energy storage technologies encompass a wide range of types with varying functions, levels of technological maturity, and economic viability [14]. Each storage technology possesses distinct advantages and limitations, accompanied by a set of technical barriers and economic challenges. Among them, solid–liquid phase-change material (PCM) storage stands out due to the intrinsic safety, high storage capacity, and low cost. When integrated with a new-type power system, the technology demonstrates significant application potential and provides effective support to the grid [15].

PCMs, due to their unique phase transition characteristics, enable efficient and reversible energy storage, offering notable advantages. PCMs have increasingly become part of the mainstream storage landscape, alongside electrochemical storage, gravitational energy storage, and compressed air energy storage [16,17]. The application scenarios of PCMs are diverse: serving as an “energy buffering barrier” for thermal runaway protection in electric vehicle batteries, functioning as an “optical camouflage coating” for infrared stealth, and even offering potential as future “information storage media” [18,19,20].

Within power systems, PCMs are also regarded as promising “dynamic energy reservoirs” for large-scale energy regulation and storage. However, the widely used solid–liquid PCM systems face two major technical bottlenecks. First, the commonly observed leakage of the liquid phase during the transition process complicates material encapsulation and introduces safety risks, severely hindering commercialization [21,22,23]. Second, the inherently low thermal conductivity of conventional pure PCMs leads to significant thermal resistance during charging and discharging processes, substantially reducing overall system efficiency [24]. Such limitations pose critical obstacles to the breakthrough development of PCM-based energy storage technologies [25,26,27].

Guar gum, extracted from guar beans, possesses multiple advantages, including non-toxicity, renewability, biodegradability, and low cost. It is commonly employed as a polysaccharide-based thickener and would form a three-dimensional (3D) framework structure after water absorption, followed by freeze-drying [28]. With continuous performance optimization, the bio-based guar gum framework encapsulation technology has gradually matured. The resulting porous structure is customizable, and the framework itself is lightweight, making it fully suitable as a 3D, porous, interconnected encapsulation matrix for coating and storage of PCMs. By selecting specific substances to modify the framework, the compatibility among raw materials would be enhanced, effectively addressing the leakage issue and expanding its application scope. Polyethylene glycol (PEG) features strong chemical stability, environmental friendliness, high energy storage density, and low supercooling [29]. Its phase-change temperature can be adjusted by varying the molecular weight, allowing it to accommodate a broader range of application scenarios. These characteristics make PEG highly suitable for industrial-scale production and widely used in the field of energy storage materials [30].

Currently, SiO_2_ aerogel has attracted considerable attention as a thermal storage framework material, owing to the ease of preparation, inherent non-flammability, high surface area, elevated porosity, and ultralight density [31]. The sol–gel method, recognized as a classical and convenient approach for preparing SiO_2_ aerogels, faces challenges in precisely controlling the water-to-silicon ratio during practical operation, which directly affects the properties of products and represents a major technical bottleneck in the fabrication. To address the issue of poor thermal conductivity in PCMs, titanium dioxide (TiO_2_) has been proven to boost heat transfer [32]. Its non-toxic nature preserves environmental friendliness, making it an excellent and widely validated solution.

In this study, a high-performance composite phase-change material (PCM) was ingeniously designed and constructed, showcasing significant innovation. A porous structure derived from the guar gum was utilized as the macroscopic encapsulation framework, while a self-prepared silica aerogel with a high specific surface area acted as the microscopic encapsulation framework. By integrating and synergistically encapsulating these dual frameworks, a three-dimensional (3D) porous structure GS was successfully developed. To augment the thermal conductivity of the material, titanium dioxide (TiO_2_) was introduced, resulting in the optimized thermally enhanced encapsulation framework (GTS). Subsequently, polyethylene glycol (PEG) was impregnated into this structure via vacuum impregnation, leading to the formation of composite PCM (GTS-PEG). This novel material not only exhibits excellent leakage resistance and superior thermal conductivity but also demonstrates highly efficient energy storage and conversion capabilities. When integrated with long-duration power auxiliary services, the material is expected to enable efficient conversion between electrical and thermal energy through an electric heating device connected to the power grid. To date, no studies have documented a composite supporting framework that integrates SiO_2_ and guar gum while simultaneously incorporating TiO_2_ as a thermal conductivity enhancer in PCMs. Extending the design concept to grid-scale energy storage presents an even more forward-looking and innovative prospect.

## 2. Experimental

### 2.1. Materials

Guar gum was purchased from Macklin Inc. (Shanghai, China). Nano titanium dioxide (nano-TiO_2_) was commercially supplied by Aladdin Industrial Inc. (Shanghai, China). Tetraethyl orthosilicate (TEOS) was procured from Guangzhou Chemical Reagent Factory (Guangzhou, China). Ethanol absolute (C_2_H_6_O) and Hydrochloric acid solution (HCl) were acquired from Lingfeng Chemical Reagents Co., Ltd. (Shanghai, China). Polyethylene glycol (PEG) was obtained from Aladdin Industrial Inc. (Shanghai, China). All the reagents were used directly without any further treatment.

### 2.2. Preparation of SiO_2_ Aerogel

Initially, TEOS, C_2_H_6_O, and deionized water were combined in an optimized proportion and stirred for 0.5 h using a mechanical stirring paddle. Subsequently, HCl was gradually added to adjust the pH to between 2 and 3, followed by continued mechanical stirring for 1.5 h. NH_3_·H_2_O was then introduced to raise the pH to 10. The resulting solution was aged for 6 h, after which it underwent vacuum freeze-drying for 72 h to yield sheet-like SiO_2_ aerogel particles.

### 2.3. Preparation of GTS

To begin with, 0–5 g of hydrophilic nano-TiO_2_ was dispersed in 60 mL of deionized water and magnetically agitated for 1 h. Next, 0.5 g of guar gum was dissolved in 60 mL of the above solution and mixed at 25 °C for 8 h. Afterwards, a designated amount of SiO_2_ aerogel was introduced into the mixture and blended mechanically for 1.5 h. The resulting solution was then poured into a mold and subjected to vacuum freeze-drying for 48 h to form the GTS with a 3D network structure and enhanced thermal conductivity. In particular, if SiO_2_ aerogel was added in the experiment without the addition of TiO_2_, the resulting framework was designated as GS. Conversely, if TiO_2_ was added without SiO_2_, the resulting framework was designated as GT.

### 2.4. Preparation of GTS-PEG Composite PCMs

**Figure 1:**
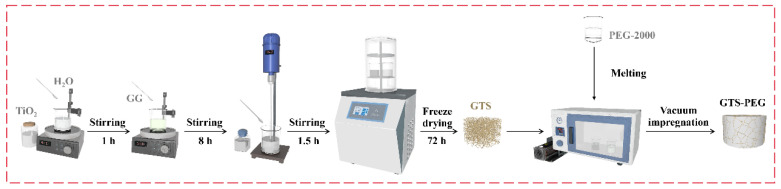
The fabrication process of GTS-PEG composite PCMs.

The GTS-PEG composite PCMs were prepared using a vacuum impregnation technique. GTS was immersed in molten PEG at 80 °C within a vacuum heating oven for 1 h. Afterward, excess PEG on the surface was removed using filter paper, yielding composite PCM: GTS-PEG. To provide a clearer understanding of the entire preparation process, a schematic illustration is shown in Figure 1. Notably, the composite PCMs obtained by applying the same process to the GS and GT frameworks described above are designated as GS-PEG and GT-PEG, respectively. It is also worth noting that GTS-PEG-x represents the amount of TiO_2_ added, where x = 0–5 indicates the addition of 0 to 5 g. For example, GTS-PEG-3 represents the TiO_2_ addition of 3 g.

### 2.5. Characterization

The specific surface areas were measured with a surface area analyzer (ASAP2460, Norcross, GA, USA) and determined using the Brunauer–Emmett–Teller approach. The microstructure of the sample was examined using a scanning electron microscope (Hitachi SU8010, Tokyo, Japan) operated at an acceleration voltage of 3 kV. The chemical structure was analyzed using a Fourier transform infrared spectrometer (Nicolet iS50, Madison, WI, USA), while the crystal structure was examined by X-ray diffraction (PANalytical B.V. X’pert Powder, Almelo, The Netherlands). The thermal conductivities were recorded using a Hot Disk apparatus (TPS2500, Göteborg, Sweden), and the testing temperature was maintained at 25 °C, with an accuracy of ±2% for thermal conductivity. The instrument employs a nickel-alloy double-spiral probe that serves as both the heat source and temperature sensor, encapsulated by a polyimide protection layer. During measurement, the probe is placed between two samples of identical size to form a sandwich structure. A constant current is applied to induce a temperature rise, and thermal properties are obtained by analyzing the recorded heat transfer process. The phase-change latent heat was investigated using a differential scanning calorimeter (TA Q20, New Castle, DE, USA) under a heating/freezing rate of 10 °C/min. The real-time temperature data were collected automatically using an Agilent 34970A data collector (Santa Rosa, CA, USA) connected to thermocouples. The experimental procedure for obtaining the step-cooling curve was as follows: A total of 10.0 g of the composite PCM was weighed and placed into a quartz tube. A K-type thermocouple (accuracy: 0.1 °C) was inserted into the center of the sample for data acquisition, and the tube was subsequently sealed with a rubber stopper. The tube was then immersed in a digitally controlled thermostatic water bath at 65 °C. After the sample was maintained at this temperature for 30 min, the quartz tube containing the thermocouple was rapidly transferred into another water bath at 25 °C, and the temperature variation was recorded in real time. Throughout the experiment, the Agilent 34970A data acquisition system (Santa Rosa, CA, USA) was employed to capture the temperature–time profile of the composite PCM, from which the step-cooling curve was plotted.

## 3. Discussion and Results

### 3.1. Optimized Preparation of SiO_2_ Aerogel

**Figure 2:**
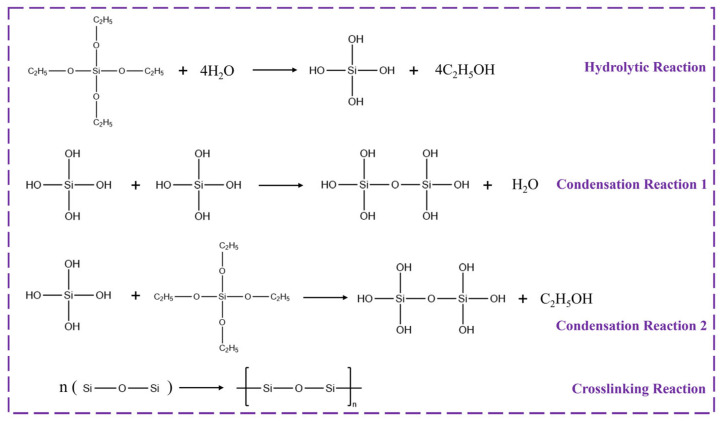
Process diagram of sol–gel method [33,34].

In the experiment, tetraethyl orthosilicate (TEOS) was selected as the silicon source, and SiO_2_ aerogel was prepared via the sol–gel method. Specifically, the preparation process primarily involves three steps: sol–gel transition, aging, and drying. Among them, the sol–gel process refers to the hydrolysis and condensation of TEOS under acidic conditions, which subsequently leads to the formation of a 3D network structure. Furthermore, a schematic illustrating the entire process is shown in the Figure 2 below.

The addition of hydrochloric acid (HCl) solution in the process serves to catalyze the hydrolytic reaction, while ethanol acts as a co-solvent to ensure a homogeneous and stable reaction environment. Sodium hydroxide (NaOH) is introduced to promote the condensation reaction within the system. As one of the key reactants, the amount of water plays a critical role in determining the morphology and properties of the final product. To ensure complete hydrolysis, the higher water content is generally desirable. On the other hand, excessive water will inhibit the subsequent condensation of hydrolyzed Si–OH groups, thereby affecting the structural formation of SiO_2_ aerogel. Based on experimental results, the differences in specific surface area and pore size of SiO_2_ aerogels prepared with varying water contents are summarized in Table 1.

The preparation of a high-specific-surface-area micro-framework structure is key to effectively attaching PCMs and enhancing the system’s leakage resistance. In the experiment, samples labeled 1, 2, 4, and 5 represent TEOS to water molar ratios of 1:2, 1:4, 1:5, 1:6, and 1:8, respectively. Observational data reveal that the highest specific surface area of SiO_2_ aerogels produced by these five samples was 476.14 m^2^/g (TEOS:H_2_O = 1:5), while the lowest was 262.76 m^2^/g (TEOS:H_2_O = 1:2). The underlying mechanism can be analyzed in conjunction with the reaction process: As illustrated in the hydrolysis step shown in Figure 2, one TEOS molecule requires at least 4 H_2_O molecules for complete hydrolysis (to form Si(OH)_4_). When water molecules are moderately excessive, it not only ensures the full progress of the hydrolysis reaction but also accelerates the reaction kinetics. Additionally, an appropriate increase in water content facilitates sufficient contact between the subsequently formed SiO_2_-gel network and the solvent, leading to a more expanded gel structure and effectively avoiding particle agglomeration or network overlap. After vacuum freeze-drying, a porous structure with a high specific surface area is formed. However, excessive water molecules will inhibit the subsequent condensation reaction 1, resulting in insufficient formation of siloxane bonds (Si-O-Si), which, in turn, impairs the structural integrity and specific surface area of the aerogel. Therefore, it is necessary to screen the equilibrium water content through experiments and ultimately determine that a TEOS:H_2_O ratio of 1:5 is the optimal proportion that balances the sufficiency of hydrolysis and the efficiency of condensation reactions. In fact, the initial stage involved exploratory experiments using gradient TEOS:H_2_O ratios (1:2, 1:4, 1:6, and 1:8), and the samples prepared at ratios of 1:4 and 1:6 were found to already exhibit relatively high specific surface areas. To more precisely determine the optimal range, an additional sample with a 1:5 ratio was prepared. The results confirmed that the specific surface area reached the peak, thus identifying this ratio as the optimal reaction condition.

**Figure 3:**
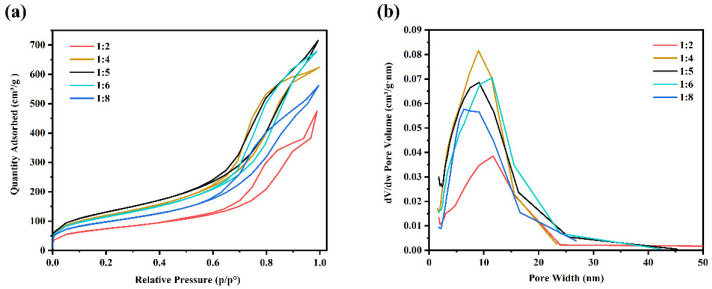
Different molar ratio (TEOS:H_2_O) samples: (**a**) nitrogen adsorption–desorption isotherms, and (**b**) the pore size distribution curve.

The specific surface area and pore size test results for samples with different TEOS:H_2_O molar ratios are presented in Figure 3a. The adsorption–desorption isotherms exhibit a reversible closed loop, resembling a Type IV isotherm, and fail to reach equilibrium at pressures near the saturation vapor pressure. Such behavior indicates the presence of slit-like pores formed by plate-like particles. From the perspective of PCM encapsulation, the resulting product requires both a high specific surface area to optimize performance and an appropriate pore size to store a portion of the PCM while retaining insulating air through capillary forces. Experimental results revealed that when TEOS:H_2_O = 1:2, the pore size was excessively large, suggesting that insufficient precursor content led to poor formation of the crosslinked network. As depicted in Figure 3b, the sample with a TEOS:H_2_O ratio of 1:5 displayed a moderate pore size, ranging from 2 to 50 nm, which is indicative of a mesoporous structure. Accordingly, this ratio was adopted in subsequent experiments to prepare aerogels with optimal performance.

### 3.2. Relevant Mechanisms of Action

**Figure 4:**
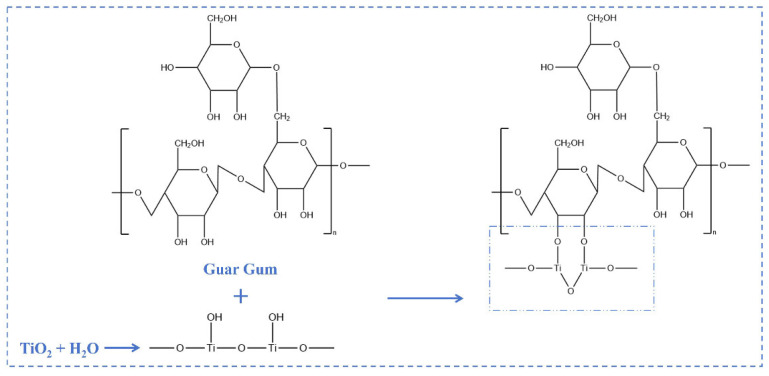
Schematic diagram of interaction mechanism between TiO_2_ nanoparticles and guar gum [35,36,37].

The corresponding mechanism between TiO_2_ and guar gum is illustrated in Figure 4. After hydrolysis treatment, the surface of TiO_2_ molecules becomes enriched with hydroxyl groups. These hydroxyl groups readily undergo complexation reactions upon contact with hydroxyl groups on the surface of guar gum. Subsequently, a relatively stable composite structure forms through vacuum freeze-drying. In addition, due to the strong hydrogen-bonding capability of hydroxyl groups, they readily interact with highly electronegative oxygen atoms (O) to form hydrogen bonds, including those between the matrix and PEG within the PCM. Both the hydrogen-bonding interactions and the complexation reactions among hydroxyl groups facilitate the formation of a more robust and stable composite system.

### 3.3. Microstructural Characterization

**Figure 5:**
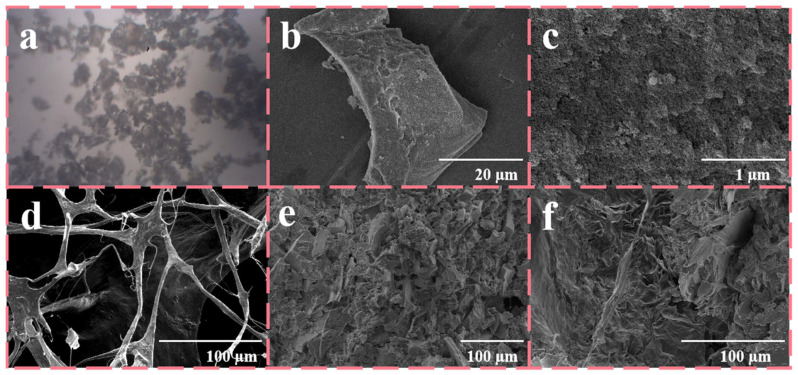
SEM micrographs of (**a**) the image of flaky SiO_2_ aerogel under optical microscopy, (**b**) flaky SiO_2_ aerogel, (**c**) flaky SiO_2_ aerogel surface, (**d**) guar gum skeleton, (**e**) GTS framework, and (**f**) GTS-PEG.

The microscopic structural morphology is presented in Figure 5, which initially shows the optical microscopy (a) and scanning electron microscopy (SEM) images of SiO_2_ aerogel (b), respectively, offering additional insight into the microscale structure and morphological features of microsheets. Simultaneously, Figure 5c illustrates the rough surface of SiO_2_ aerogel microsheets. This surface texture not only facilitates the attachment and solidification of liquid PCMs but also provides nucleation sites, thereby effectively reducing the degree of supercooling during the solidification process.

Next, Figure 5d presents the skeletal microscopic morphology of pure guar gum after vacuum freeze-drying treatment. It can be observed that the network presents uneven hole sizes and inconsistent thickness of the framework. Although the structure provides large-scale storage space for PCM, its relatively low surface area results in difficulty in achieving sustained attachment of the PCM. Figure 5e presents skeletal microscopic morphology after adding a large amount of SiO_2_ aerogel microsheets and TiO_2_ particles. The microsheets are loosely distributed within the framework. This mode of incorporation markedly increases the surface area of the skeleton, thereby increasing the attachment points for PEG, which provides more favorable conditions. At the same time, Figure 5f displays the microscopic morphology of the composite PCM after adsorbing PEG. It is clear that the raw materials are tightly integrated and fused together to form a whole. The original guar gum skeletal structure remains faintly visible, suggesting that the composite process successfully combines components.

The microscopic structural morphology is presented in Figure 5, which initially shows the skeletal microscopic morphology of pure guar gum after vacuum freeze-drying treatment. It can be observed that the network presents uneven hole sizes and inconsistent thickness of the framework. Although the structure provides large-scale storage space for PCM, its relatively low surface area results in difficulty in achieving sustained attachment of the PCM. Figure 5b presents skeletal microscopic morphology after adding a large amount of SiO_2_ aerogel microsheets and TiO_2_ particles. The microsheets are loosely distributed within the framework. This mode of incorporation markedly increases the surface area of the skeleton, thereby increasing the attachment points for PEG, which provides more favorable conditions.

At the same time, Figure 5c displays the microscopic morphology of the composite PCM after adsorbing PEG. It is clear that the raw materials are tightly integrated and fused together to form a whole. The original guar gum skeletal structure remains faintly visible, suggesting that the composite process successfully combines components. Figure 5d,e present the optical microscopy and scanning electron microscopy (SEM) images of SiO_2_ aerogel, respectively, offering additional insight into microscale structure and morphological features of microsheets. Simultaneously, Figure 5f illustrates the rough surface of SiO_2_ aerogel microsheets. This surface texture not only facilitates the attachment and solidification of liquid PCMs but also provides nucleation sites, thereby effectively reducing the degree of supercooling during the solidification process.

### 3.4. Macroscopic Characterization

**Figure 6:**
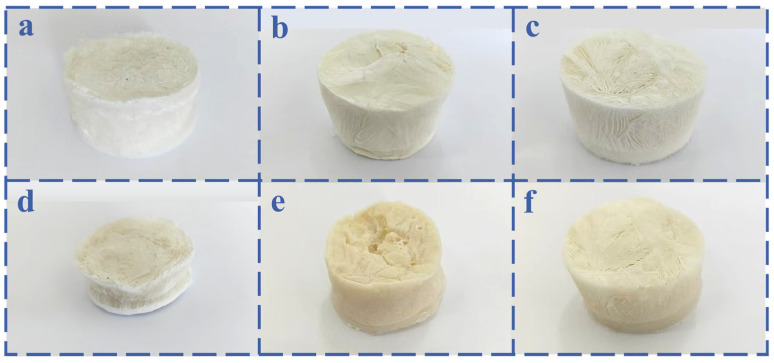
Digital photo images of the obtained frames: (**a**) guar gum skeleton, (**b**) GT framework, (**c**) GTS skeleton frame, composite PCMs, (**d**) the composite PCM created by adsorbing PEG into the guar gum framework, (**e**) GT-PEG, and (**f**) GTS-PEG.

The macroscopic morphology of the materials is illustrated in Figure 6. Figure 6a depicts the guar gum skeleton after vacuum freeze-drying, exhibiting a fluffy and soft structure with ample internal space for storing PCM. However, the overall framework is relatively fragile, and after absorbing the PCM, significant shrinkage and collapse are observed, as shown in Figure 6d. The introduction of TiO_2_ nanoparticles fails to alleviate the structural collapse, as illustrated in Figure 6e. Notably, when SiO_2_ aerogel is incorporated into the system, the composite reveals a robust and stable structure, as shown in Figure 6f. The results above clearly demonstrate the necessity of incorporating SiO_2_ aerogel into this system.

### 3.5. Thermal Conductive Properties of GTS-PEG

**Figure 7:**
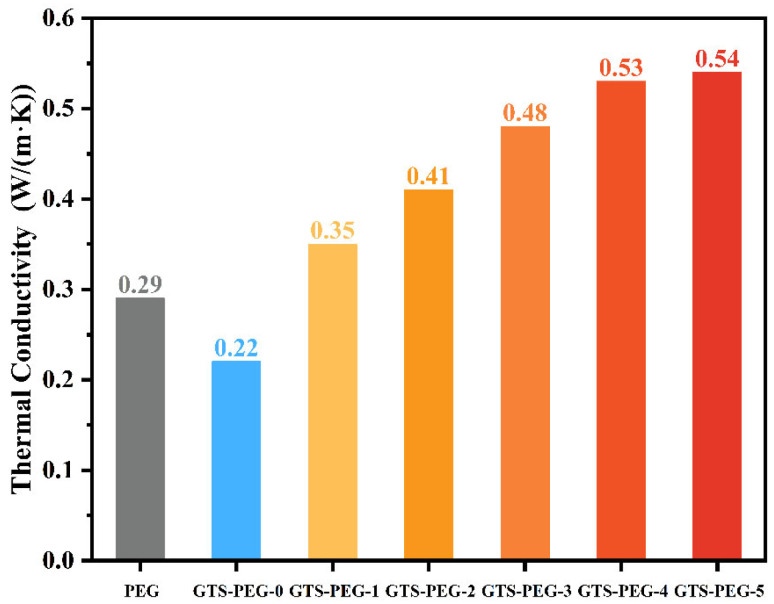
Thermal conductivities of pure PEG and GTS-PEG.

To more clearly illustrate differences in thermal conductivity, the transient plane source (TPS) method was employed for precise measurements. As shown in Figure 7, the thermal conductivity of pure PEG is 0.29 W/(m·K). After the introduction of guar gum and SiO_2_ framework, the overall thermal conductivity decreased to 0.22 W/(m·K), which is attributed to the inherently low thermal conductivity of the supporting structure. In addition, the incorporation of other substances increased the complexity of the system, which further enhanced phonon scattering during heat transfer [38]. By contrast, upon the inclusion of nano-TiO_2_ particles, the thermal conductivity of the samples indicated a marked improvement. Specifically, the thermal conductivities of GTS-PEG-1, GTS-PEG-2, GTS-PEG-3, GTS-PEG-4, and GTS-PEG-5 were measured as 0.35 W/(m·K), 0.41 W/(m·K), 0.48 W/(m·K), 0.53 W/(m·K), and 0.54 W/(m·K), respectively. Compared to GTS-PEG-0, the thermal conductivity of GTS-PEG-4 increased by 140%, and that of GTS-PEG-5 increased by 145%. The observed data demonstrate that TiO_2_ plays a significant role in enhancing thermal conductivity. With increasing TiO_2_ content, the thermal conduction network becomes denser and more efficient [13]. Considering both cost and performance, GTS-PEG-4 was selected as the target for subsequent investigations. Unless otherwise stated, “GTS-PEG” hereafter refers to “GTS-PEG-4”.

### 3.6. The Surface Structure of the GTS Framework

**Figure 8:**
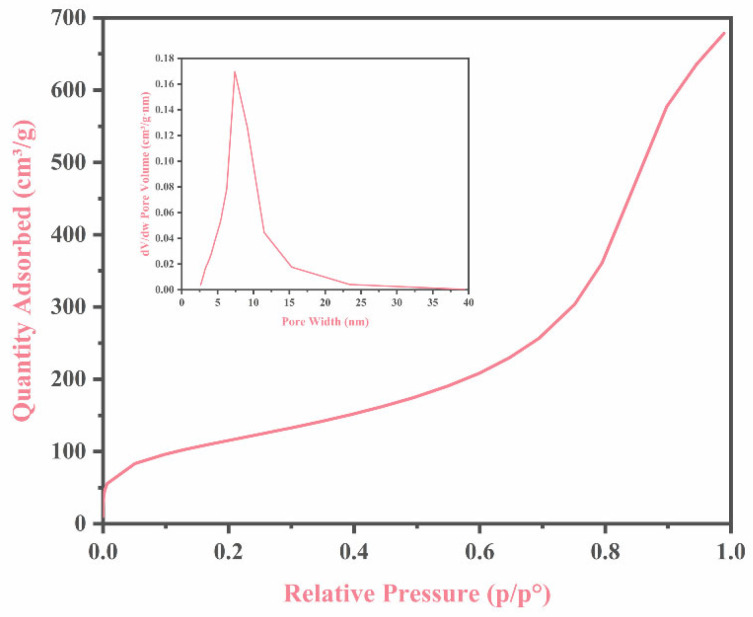
Nitrogen adsorption–desorption isotherms and the pore size distribution curve of the prepared GTS framework.

The nitrogen adsorption–desorption isotherm of the composite framework is presented in Figure 8, which demonstrates a typical Type II characteristic. Such a pattern indicates that the adsorption process within the structure is primarily dominated by free and uniform multilayer reversible adsorption. Specifically, once monolayer adsorption reaches completion, the second adsorption layer begins to form gradually with increasing relative pressure. As the pressure approaches the saturation vapor pressure, the number of adsorption layers continues to increase. Measurements show that the specific surface area of the composite framework is 404.52 m^2^/g, and the pore size is 9.48 nm. Based on pore diameter, the predominant pore type on the surface is mesopores. This structural characteristic suggests that the composite skeleton, when used as an adsorbent, is able to provide abundant attachment space and active sites for adsorbates, revealing excellent adsorption potential.

### 3.7. Crystal Structure of the GTS Framework

**Figure 9:**
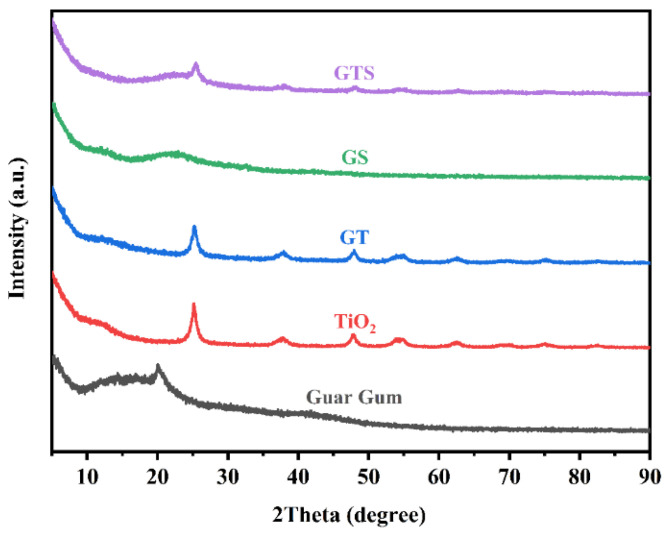
XRD patterns of guar gum, TiO_2_, GT, GS, and GTS framework.

In XRD patterns, the guar gum profile indicates a distinctly amorphous structure, characterized by a broad, weak peak (Figure 9). The TiO_2_ diffraction pattern reveals typical peaks at 2θ = 25.2°, 38.0°, 47.9°, 54.0°, 55.1°, and 62.7°, corresponding to the (101), (004), (200), (105), (211), and (204) crystal planes of the anatase phase, in that order. The XRD patterns of GT, GS, and GTS demonstrate diffraction peaks that reflect the superposition of the individual crystalline phases of each raw material, with only slight changes in peak intensities and no new peaks appearing. It can be inferred that, despite the coordination process between the hydroxyl groups of guar gum and hydrated TiO_2_, there is no significant alteration in the original crystal structures of the raw materials. Furthermore, the materials are well-integrated in a physical form, which facilitates the establishment of a strong thermal conduction network.

### 3.8. Analysis of GTS-PEG Heat Storage and Release Properties

**Figure 10:**
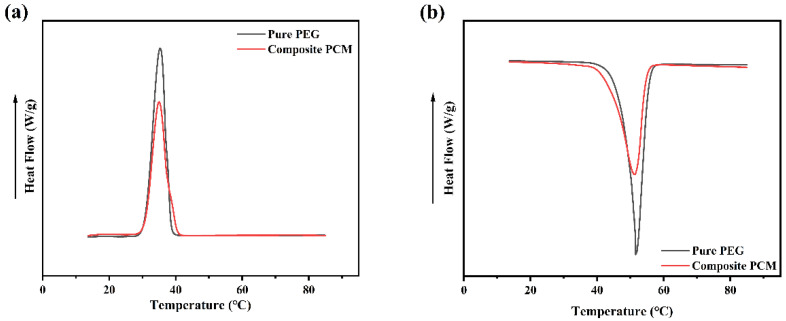
DSC curves, cooling curves (**a**), and heating curves (**b**) of pure PEG and GTS-PEG.

Differential scanning calorimetry (DSC) test results display (Figure 10) that after adding the guar gum–TiO_2_-silica (GTS) composite framework, the initial melting temperature (T_onset_) decreased by 4.27 °C (43.65 °C compared to 47.92 °C of pure PEG), suggesting that the introduction of the GTS framework significantly improved the thermal conductivity of the system. Such an enhancement is further validated during the solidification process: under the same cooling rate, the composite material processes a slightly higher solidification temperature compared to pure PEG, indicating that the GTS framework facilitates an efficient thermal conduction pathway and promotes phonon transfer. Specific thermodynamic parameters are as follows: pure PEG system—melting phase-change enthalpy: 195.11 J/g, solidification phase-change enthalpy: 186.01 J/g; GTS–PEG composite system—melting phase-change enthalpy: 164.16 J/g, solidification phase-change enthalpy: 151.07 J/g. These data suggest that the addition of the GTS framework elevates the thermal conductivity of the system while keeping the phase-change enthalpy within a reasonable range, validating the effectiveness of the composite structure in improving heat transfer efficiency.

Furthermore, the results demonstrate that the melting enthalpies of GTS-PEG-0, GTS-PEG-1, GTS-PEG-2, GTS-PEG-3, and GTS-PEG-5 are 174.96, 172.18, 169.40, 166.63, and 155.12 J/g, respectively, while the corresponding solidification enthalpies are 161.01, 158.45, 155.90, 153.35, and 142.76 J/g. Although the nano-TiO_2_ particles do not participate in the phase-change and are introduced to improve thermal conductivity—thereby causing a slight reduction in phase-change enthalpy—the overall enthalpy values remain satisfactory.

### 3.9. Temperature–Time Variation During Cooling Processes of GTS-PEG

**Figure 11:**
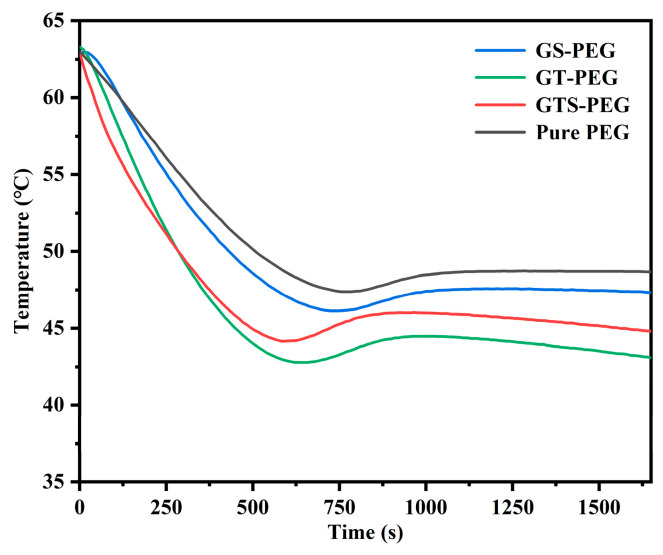
Temperature–time cooling curves of the GTS-PEG.

The analysis of the step-cooling curve clearly reveals the energy release performance of GTS-PEG under working conditions (Figure 11). The incorporation of additives significantly promotes thermal release efficiency of the composite PCM. Compared with the pure PEG system, the cooling time from 65 °C to the phase transition point is substantially reduced. Upon further incorporation of nano-TiO_2_ particles, the thermal conductivity of the composite material is further improved, confirming the cooperative contribution of the GTS framework and indicating that the framework structure establishes thermal conduction pathways to facilitate phonon transport. It is worth noting that although the GT-PEG system delivers better cooling heat transfer efficiency than the GTS framework, the structural support provided by SiO_2_ aerogel remains indispensable. Despite relatively low thermal conductivity and a slight reduction in overall heat transfer performance, it still satisfies the requirements for conventional applications. Importantly, the supercooling of GTS–PEG is only 1.82 °C, which is sufficiently low to be considered negligible, demonstrating its advantageous efficiency under practical operating conditions.

### 3.10. The Leakage-Proof Performance of GTS-PEG

**Figure 12:**
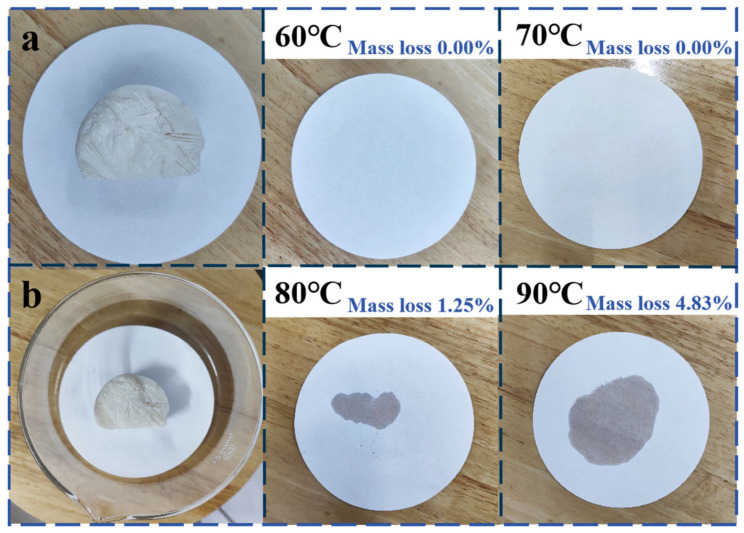
The leakage-proof performance of GTS-PEG. (**a**) Bulk GTS–PEG placed on filter paper. (**b**) Bulk GTS–PEG together with the filter paper placed in a glass dish and subsequently transferred to a drying oven.

The leakage performance of the PCM is visually presented in Figure 12. In the experiment, the sample was placed in an oven and continuously heated for 30 min at temperatures of 60 °C, 70 °C, 80 °C, and 90 °C. Directly placing the bulk PCM on filter paper for visual inspection may lead to misjudgment, because the leaked liquid PCM can be concealed by the solid material itself, producing an apparent “no-leakage” result. Therefore, the leakage extent was assessed based on the change in sample mass and the degree of impregnation observed on the filter paper beneath the sample. Data analysis confirmed that the mass proportion of leakage relative to the original total mass is 0.00%, 0.00%, 1.25%, and 4.83%, in sequence with temperature rise. Combined with previous DSC test findings, the working temperature range of the PCM is roughly defined as 20 °C to 60 °C. Overall, the experimental results and analysis indicate that when the environmental temperature is at or slightly above the material’s working temperature range, the composite PCM demonstrates excellent thermal stability, effectively suppressing leakage and ensuring reliable performance.

## 4. Conclusions

In this work, biomass-based guar gum was used as the primary framework, and self-prepared SiO_2_ aerogel microsheets served as the secondary framework. The dual-scale network synergistically cooperated to encapsulate the PEG phase-change material (PCM), with TiO_2_ nanoparticles added as a thermal conductivity enhancer. By fine-tuning the silica-to-water ratio during the preparation process, the SiO_2_ aerogel with a peak specific surface area of 473.21 m^2^/g was successfully obtained and integrated into the system as a microscopic support structure. The resulting composite PCM, designated as GTS-PEG, exhibits a robust skeleton, as confirmed by microstructural characterization and macroscopic design analysis, allowing for substantial PEG storage. Thermal conductivity testing revealed a 140% increase in heat transfer efficiency of GTS-PEG-4 compared to the sample prior to the addition of TiO_2_ particles, reaching 0.53 W/(m·K), due to the construction of continuous heat transfer pathways. In addition, differential scanning calorimetry results demonstrated strong energy storage capability, with melting and solidification enthalpies of 164.16 J/g and 151.07 J/g, respectively. After continuous heating at 90 °C for 30 min, the maximum mass loss due to leakage was only 4.83% of the initial mass. The findings above confirm that GTS-PEG possesses excellent thermal conductivity, heat storage capacity, and leakage resistance, enabling efficient thermal energy storage and release. The performance parameters of the composite PCMs—including thermal behavior, encapsulation stability, and environmental compatibility—were benchmarked against those reported in previous studies, as summarized in Table S1. The GTS–PEG system exhibits outstanding, notable eco-friendly properties, as its constituent materials (SiO_2_ aerogel, guar gum, and TiO_2_) are non-toxic, non-irritating, and commonly employed in daily necessities. At the same time, it maintained balanced overall performance, setting it apart from many other PCMs.

Going forward, the composite PCM will be integrated with appropriate devices to form independent and efficient energy storage systems, addressing challenges of grid fluctuations caused by large-scale integration of variable renewable energy sources. On the generation side, deploying corresponding phase-change storage systems is able to enhance the utilization efficiency of renewable power; on the grid side, such systems help ensure safe and efficient operation; and on the user side, the conversion between electrical and thermal energy may reduce thermal costs and improve autonomy, demonstrating broad application potential.

## Figures and Tables

**Table 1:** The specific surface area and pore size of SiO_2_ aerogels generated with different raw material ratios.

Number	TEOS	H_2_O	Absolute Alcohol	Specific Surface Area (m^2^/g)	Average Pore Diameter (nm)
1	1	2	4	262.76	11.03
2	1	4	4	428.44	8.11
3	1	5	4	476.14	8.36
4	1	6	4	418.15	9.59
5	1	8	4	343.79	8.29

## Data Availability

The data that support the findings of this study are available from the corresponding author upon reasonable request.

## Supplementary Materials

The following supporting information can be downloaded at https://www.mdpi.com/article/10.3390/nano15241887/s1, Table S1. Comparison of various properties in this work with literature reports. Refs. [39,40,41,42,43,44,45,46,47] are cited the supplementary material file.
