# Supplementary material for: A Dual-Scale Encapsulation Strategy for Phase Change Materials: GTS-PEG for Efficient Heat Storage and Release
Source: Nanomaterials (Basel). 2025 Dec 16;15(24):1887. doi: 10.3390/nano15241887 (PMC12736329; doi:10.3390/nano15241887)
Supplement: Supplementary file 1 [file nanomaterials-15-01887-s001.zip › nanomaterials-3961140-supplementary.pdf]

Supporting Information

A Dual-Scale Encapsulation Strategy for Phase Change Materials: GTS-PEG for Efficient Heat Storage and Release

Sixing Zhang [1], Guangyao Zhao [1*], Zhen Li [1], Zhehui Zhao [1], Jiakang Yao [1], Geng Qiao [2], Zongkun Chen [2], Yuwei Wang [3], Donghui Zhang [3], Dongliang Guo [4], Zhixiang Zhu [2*] and Yu Han [1*]

1.State Key Laboratory of Advanced Power Transmission Technology, China Electric Power Research Institute Co., Ltd., Beijing 102209, China;
2.Global Energy Interconnection Research Institute Europe GmbH, Berlin 10623, Germany;
3.Beijing Guodian Futong Science and Technology Development Co.,Ltd., Beijing 100070, China;
4.State Grid Jiangsu Electric Power Co., Ltd., Research Institute, Nanjing, 211103, China.
* Correspondence: zhaoguangyao@epri.sgcc.com.cn

Table S1. Comparison of various properties in this work with literature reports.

PCM	Thermal conductivity (W/(m·K))	Mass percentage of phase change substances (%)	Environmentally benign properties	Reference
Paraffin Wax	0.45	78	1	[1]
Paraffin Wax	0.26	72	1	[2]
Paraffin Wax	0.78	74	1	[3]
PEG	0.44	75	1	[4]
PEG	0.40	96	3	[5]
PEG	0.47	-	1	[6]
m-Erythritol	0.76	56	3	[7]
Shellac Wax	0.33	100% No encapsulation	5	[8]
n-Octadecane	0.45	50	3	[9]
PEG	0.54	84	4	This work

The term "Proportion of phase change substances" refers to the mass percentage of components that participate in the phase transition within the synthesized composite PCM. The "Environmentally benign properties" are assessed according to both the source materials and the fabrication procedure, using a five-level rating system in which 5 denotes the highest score and 1 the lowest. As shown in the table, the composite PCM developed in this work demonstrates well-balanced and superior overall performance for thermal energy storage.

Reference:

[1] C. Feng, K. Sun, J. Ji, G. Cui, L. Hou, M. Shi, F. Wei, W. Yang. Flexible phase change materials for overheating protection of electronics. *Journal of Energy Storage* **2024**, 84, 110719.

[2] X. Kong, Y. Fu, J. Yuan. Novel flexible phase change materials with high emissivity, low thermal conductivity and mechanically robust for thermal management in outdoor environment. *Applied Energy* **2023**, 348, 121556.

[3] X. Zhang, J. Yuan, X. Kong, J. Han, Y. Shi. Coupling of flexible phase change materials and pipe for improving the stability of heating system. *Energy* **2023**, 275, 127474.

[4] D. Feng, P. Li, Y. Feng, Y. Yan, X. Zhang. Using mesoporous carbon to pack polyethylene glycol as a shape-stabilized phase change material with excellent energy storage capacity and thermal conductivity. *Microporous and Mesoporous Materials* **2021**, 310, 110631.

[5] G. Yang, B. Wang, H. Cheng, Z. Mao, H. Xu, Y. Zhong, X. Feng, J. Yu, X. Sui. Cellulosic scaffolds doped with boron nitride nanosheets for shape-stabilized phase change composites with enhanced thermal conductivity. *International Journal of Biological Macromolecules* **2020**, 148, 627-634.

[6] X. Du, J. Qiu, S. Deng, Z. Du, X. Cheng, H. Wang. Ti3C2Tx@PDA-Integrated Polyurethane Phase Change Composites with Superior Solar-Thermal Conversion Efficiency and Improved Thermal Conductivity. *ACS Sustainable Chemistry & Engineering* **2020**, 8, 5799-5806.

[7] N. Tan, T. Xie, Y. Feng, P. Hu, Q. Li, L.-M. Jiang, W.-B. Zeng, J.-L. Zeng. Preparation and characterization of erythritol/sepiolite/exfoliated graphite nanoplatelets form-stable phase change material with high thermal conductivity and suppressed supercooling. *Solar Energy Materials and Solar Cells* **2020**, 217, 110726.
[8] B.V. Rudra Murthy, V. Gumtapure. Thermo-physical analysis of natural shellac wax as novel bio-phase change material for thermal energy storage applications. *Journal of Energy Storage* **2020**, 29, 101390.
[9] C. Li, H. Yu, Y. Song, M. Wang, Z. Liu. A n-octadecane/hierarchically porous TiO2 form-stable PCM for thermal energy storage. *Renewable Energy* **2020**, 145, 1465-1473.
